# Identification and validation of QTL and their associated genes for pre-emergent metribuzin tolerance in hexaploid wheat (*Triticum aestivum* L.)

**DOI:** 10.1186/s12863-018-0690-z

**Published:** 2018-11-12

**Authors:** Roopali Bhoite, Ifeyinwa Onyemaobi, Ping Si, Kadambot H. M. Siddique, Guijun Yan

**Affiliations:** 1 UWA School of Agriculture and Environment, The University of Western Australia, Perth, WA 6001 Australia; 2 The UWA Institute of Agriculture, The University of Western Australia, Perth, WA 6001 Australia

**Keywords:** Metribuzin tolerance, Wheat, Chlorophyll, QTL, Validation, Candidate genes, LabChip®

## Abstract

**Background:**

Herbicide tolerance is an important trait that allows effective weed management in wheat crops. Genetic knowledge of metribuzin tolerance in wheat is needed to develop new cultivars for the industry. Here, we evaluated metribuzin tolerance in a recombinant inbred line (RIL) mapping population derived from Synthetic W7984 and Opata 85 over two consecutive years to identify quantitative trait loci (QTL) contributing to the trait. Herbicide tolerance was measured by two chlorophyll traits, SPAD chlorophyll content index (CCI) and visual senescence score (SNS). The markers associated with major QTL from Synthetic W7984, positively contributing to reduced phytotoxic effects under herbicide treatment were validated in two F_3/4_ recombinant inbred populations developed from crosses of Synthetic W7984 × Westonia and Synthetic W7984 × Lang.

**Results:**

Composite interval mapping (CIM) identified four QTL, two on chromosome 4A and one each on chromosomes 2D and 1A. The chromosomal position of the two QTL mapped on 4A within 10 cM intervals was refined and validated by multiple interval mapping (MIM). The major QTL affecting both measures of tolerance jointly explained 42 and 45% of the phenotypic variation by percentage CCI reduction and SNS, respectively. The identified QTL have a pure additive effect. The metribuzin tolerant allele of markers, *Xgwm33* and *Xbarc343,* conferred lower phytotoxicity and explained the maximum phenotypic variation of 28.8 and 24.5%, respectively. The approximate physical localization of the QTL revealed the presence of five candidate genes (ribulose-bisphosphate carboxylase, oxidoreductase (*rbcS*), glycosyltransferase, serine/threonine-specific protein kinase and phosphotransferase) with a direct role in photosynthesis and/or metabolic detoxification pathways.

**Conclusion:**

Metribuzin causes photo-inhibition by interrupting electron flow in PSII. Consequently, chlorophyll traits enabled the measure of high proportion of genetic variability in the mapping population. The validated molecular markers associated with metribuzin tolerance mediating QTL may be used in marker-assisted breeding to select metribuzin tolerant lines. Alternatively, validated favourable alleles could be introgressed into elite wheat cultivars to enhance metribuzin tolerance and improve grain yield in dryland farming for sustainable wheat production.

**Electronic supplementary material:**

The online version of this article (10.1186/s12863-018-0690-z) contains supplementary material, which is available to authorized users.

## Background

Infestation of broad-spectrum weeds in dry-land farming is a major yield-reducing factor (up to 50%) in wheat [1]. Metribuzin [4-amino-6-(1,1-dimethylethy1)-3-(methylthio)-1,2,4-triazin-5-(4H)-one], a triazine herbicide (group C), is a versatile herbicide that controls a wide range of weeds in dry-land farming systems [1, 2] and is registered for use in some wheat cultivars. However, most wheat cultivars lack selectivity to metribuzin due to narrow safety margins that result in crop damage. New herbicide-tolerant wheat cultivars are advantageous in the wheat industry to enhance crop protection against herbicide damage and maximize crop yield. A detailed understanding of the genetics and mechanisms of metribuzin tolerance is helpful for selection of superior wheat germplasm in breeding programs. Genetic control of metribuzin tolerance has been loosely investigated, and the genetic basis of inheritance and molecular mechanism of metribuzin tolerance in wheat is poorly understood.

Herbicide tolerance is a complex trait that could be a function of both alterations to the site of action (target-site resistance) and metabolic detoxification (non-target-site resistance) before reaching the target site [3]. Previous studies have revealed different modes of genetic control for metribuzin tolerance in crop plants. Ratliff et al. [4] reported the role of both nuclear and cytoplasmic genes in metribuzin tolerance in wheat. Villarroya et al. [5] reported that the inheritance of tolerance to metribuzin in durum wheat (*T. turgidum* L.) is a complex character involving many genes or quantitative trait loci (QTL). In contrast, for soybean, metribuzin tolerance is controlled by a single dominant gene [6]. Further, in narrow-leafed lupin (*Lupinus angustifolius* L.), Si et al. [7] reported two independent semi-dominant gene loci (*Mt3* and *Mt5*) having additive effects. Sequence analysis of the chloroplast DNA-encoded *psbA* gene and further studies based on the effect of inhibitors of cytochrome 450 monooxygenases suggested that non-target-site detoxification mechanisms may be responsible for the metribuzin tolerance phenotype. Likewise, Javid et al. [8] reported QTL for metribuzin tolerance in field pea, based on symptom scores and plant damage on a single genomic region located on linkage group IV. The gene (cytochrome P450 monooxygenase) underlying the QTL support range suggested herbicide tolerance based on non-target-site metabolism.

Metribuzin acts on photosystem II, ultimately inhibiting photosynthesis. The decline in net photosynthetic rate often reduces both chlorophyll and soluble-protein levels [9]. We reported phenotyping chlorophyll measures in wheat to assess phytotoxicity of metribuzin at the seedling stage in a previous study [10]. Chlorophyll measures correlated with grain and protein yields in winter wheat and spring barley [11, 12]. Rapid phenotyping techniques for measuring direct herbicide effects coupled with improved understanding of the genetics of such traits would speed up marker-assisted selection for herbicide tolerance breeding in wheat.

The international *Triticeae* mapping initiative (ITMI) mapping population with its high-density linkage map is expected to find QTL flanked by closely linked markers that can be readily used in breeding programs [13]. However, the plant breeding community recognizes that these putative QTL need to be validated across various genetic backgrounds before embarking upon marker-assisted selection. As a result, in this study we (a) evaluated for metribuzin phytotoxicity in ITMI mapping population derived from a cross between Synthetic W7984 and Opata 85 over two years, (b) identified QTL associated with metribuzin tolerance in the same population, (c) confirmed and validated for its genetic effect in genetic backgrounds other than the mapping population for their applicability in MAS, and (d) identified the putative candidate genes influencing metribuzin tolerance. The discovery of QTL will enhance the understanding of the intricate genetic basis of phenotype variance. These findings will provide new insights for improving wheat yields in breeding programs.

## Results

### Phenotypic evaluation of the mapping population

**Fig. 1:**
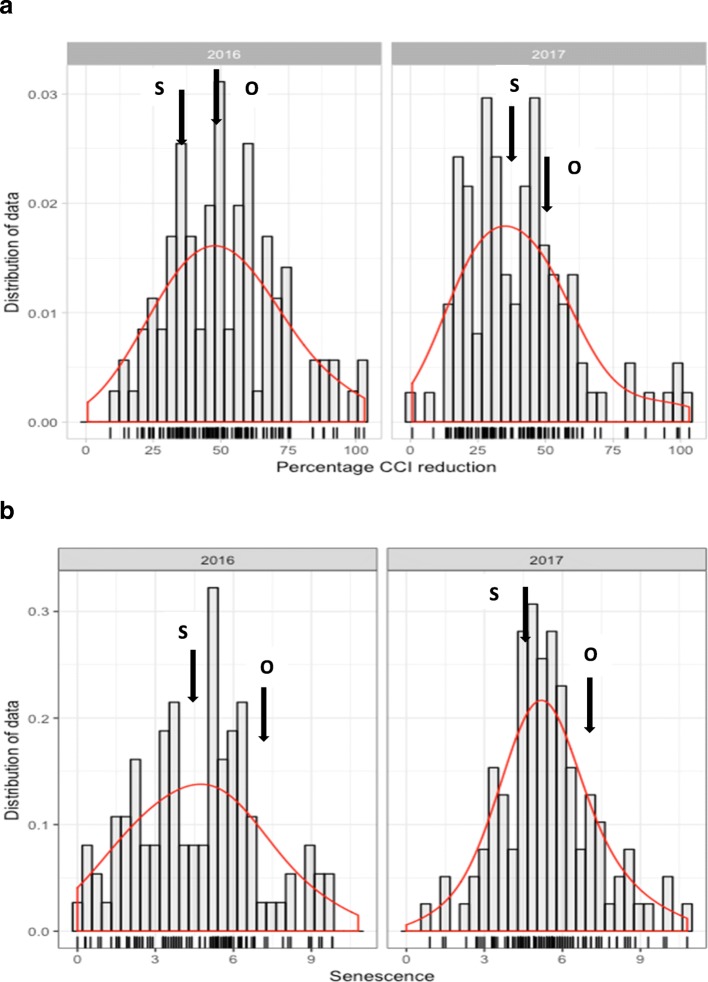
Phenotypic distribution of percentage chlorophyll content index reduction (**a**) and senescence (**b**) in Synthetic W7984 × Opata 85 RIL mapping population based on mean data measured for two years. *S* Synthetic, *O* Opata

The frequency distribution of the metribuzin reaction of the mapping population was approximately normal (Fig. 1), consistent with the polygenic control of tolerance. A wide range of phenotypic variation was present in RILs for both traits under control and metribuzin treatment conditions (Additional files 1 and 2). With metribuzin, RILs ranged from 0.5 to 37.3 and 1 to 10 using SPAD CCI and SNS, respectively. The population means remained higher than those of the parents, indicating transgressive segregations in both directions of parents. The ANOVA of phenotypic data from two years (2016 and 2017) indicated that the magnitude of differences was constant between years, genotype variance was significant (*P* ≤ 0.05), and the genotype × year interaction effect was insignificant. Therefore, average predicted values of merged two-year data were used for QTL analysis. The CCI and SNS scores had positive and significant (*P* < 0.05) correlation (data not shown). Moderate to high broad-sense heritability (*h*^2^) of 59.2 and 76.4 was estimated by CCI and SNS, respectively (Table 1).

**Table 1:** Analysis of variance for metribuzin tolerance and associated traits and their heritability estimates in the Synthetic W7984 × Opata 85 RIL mapping population measured across two years

Category	Source of variation	df	CCI	SNS
Control	Line	110	45.43**	–
	Year	1	13,217**	–
	Rep	2	3.43	–
	Year × Line	103	16.11	–
	Residual	332	11.77	–
	*h* ^2^		64.55	–
	SE		0.29	–
Metribuzin treatment	Line	110	173.94**	15.02**
	Year	1	232.23	66.58**
	Rep	2	706.55**	77.61**
	Year × Line	95	70.89	3.54
	Residual	214	90.20	5.63
	*h* ^2^		59.22	76.4
	SE		0.95	0.23

*CCI* SPAD chlorophyll content index, *SNS* senescence scale (1–10)

### QTL for chlorophyll contents

**Fig. 2:**
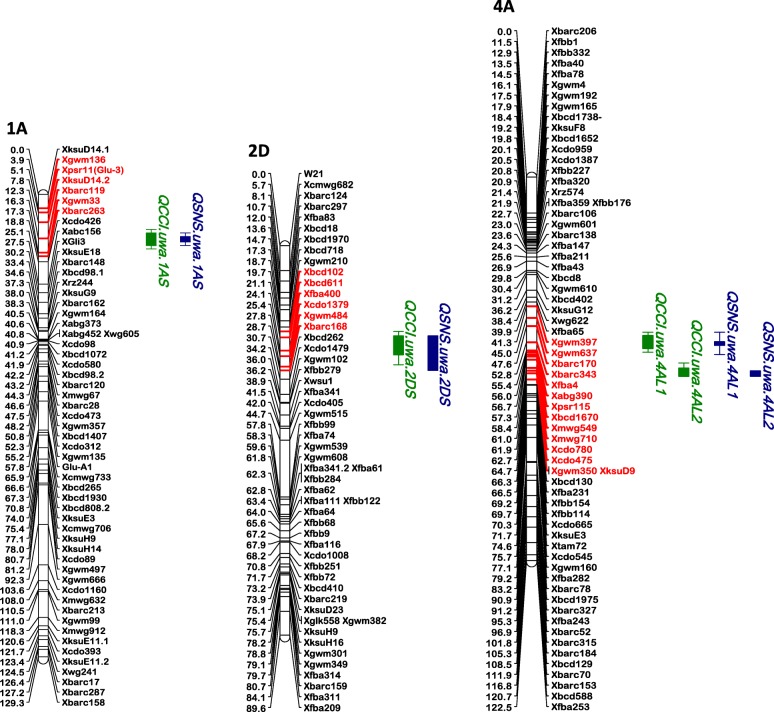
Locations of putative QTL for metribuzin tolerance in Synthetic W7984 × Opata 85 RIL population based on SPAD chlorophyll content index (CCI) reduction and senescence (SNS). QTL are indicated by solid bars and the bar length indicates a 1.0 LOD drop in the QTL support interval. Green bars indicate QTL by SPAD chlorophyll content index and blue bars indicate QTL by leaf senescence. Map distances are indicated on the left in Kosambi centimorgan and markers are indicated on the right of each chromosome. The markers in red are the flanking markers within a 1.0 LOD drop in the QTL support interval

CIM using SPAD CCI reduction and SNS detected four co-located QTL, with two on chromosome 4A and one each on chromosomes 1A and 2D (Fig. 2). The LOD scores ranged from 3.4 to 5.8 and 3 to 7.3, explaining 10–19% and 8–20% phenotypic variation by CCI reduction and SNS, respectively (Table 2). Two QTL were located on the long chromosome arm of 4A at 52.6 cM and 61.9 cM by CCI reduction and 52.8 cM and 61.9 cM by SNS. The interval location between two mapped QTL was 9.3 cM and 9.1 cM by CCI reduction and SNS, respectively, which is less than 10 cM distance. The position of multiple QTL on chromosome 4A was optimized by MIM and the estimated positions with main effects are given in Table 3. The QTL detected at position 52.6 cM and 52.8 cM contributed to large phenotypic variation with significant additive effect by CCI reduction and SNS, respectively. However, the QTL at position 61.9 cM, had decreased additive effect below the threshold value and therefore was excluded from the MIM model.

**Table 2:** Putative QTL for two phytotoxic traits (CCI reduction and senescence) affecting herbicide tolerance in ITMI Synthetic W7984 × Opata RIL population identified by composite interval mapping (CIM) at the LOD threshold ≥3

Trait	Chromosome arm	QTL	QTL position (cM)^c^	Flanking markers	CI^d^	LOD score	Additive^e^	R^2f^ (%)
CCI^a^	1 (1AS)	*Qcci.uwa.1AS*	12.3	*Xgwm136/Xgwm33*	3.9–17.3	3.4	−0.6	10
	6 (2DS)	*Qcci.uwa.2DS*	21.1	*Xgwm210/Xgwm484*	18.3–27.8	4.7	0.7	13
	10 (4AL)	*Qcci.uwa.4AL.1*	52.6	*Xbarc170/ Xbarc343*	48.6–56.7	5.8	−0.8	19
	10 (4AL)	*Qcci.uwa.4AL.2*	61.9	*Xbarc343/ Xgwm350*	59.4–63.7	4.0	−0.7	12
SNS^b^	1 (1AS)	*Qsns.uwa.1AS*	12.3	*Xgwm136/ Xgwm33*	3.9–14.3	3.0	−0.5	8
	6 (2DS)	*Qsns.uwa.2DS*	22.1	*Xgwm210.2/ Xgwm484*	18.3–29.7	7.3	0.9	20
	10 (4AL)	*Qsns.uwa.4AL.1*	52.8	*Xbarc170/ Xbarc343*	49.6–58.3	6.1	−0.8	17
	10 (4AL)	*Qsns.uwa.4AL.2*	61.9	*Xbarc170/ Xgwm350*	56.7–63.7	3.4	−0.6	10

^a^*CCI* SPAD chlorophyll content index reduction

^b^*SNS* senescence scale (1–10)

^c^QTL position from the left flanking marker (cM), within the 1-LOD support interval (CI)

^d^Support interval between the two flanking markers (cM)

^e^QTL with a negative additive effect mean alleles from the tolerant parent increase tolerance, positive additive effect mean alleles from the susceptible parent increase tolerance

^f^Proportion of phenotypic variance explained by the QTL

**Table 3:** Estimates of QTL locations by multiple interval mapping detected on the same chromosome 4AL within 10 cM distance for the two phytotoxic traits, CCI reduction and SNS

Trait	QTL	Chromosome arm	Position (cM)	Additive effect
CCI	*Qcci.uwa.4AL.1*	10 (4AL)	52.6	−0.8
	*Qcci.uwa.4AL.2*	10 (4AL)	61.9	−0.2
SNS	*Qsns.uwa.4AL.1*	10 (4AL)	52.8	−0.8
	*Qsns.uwa.4AL.2*	10 (4AL)	61.9	−0.0

### QTL effect confirmation and marker validation

In this study, two major QTL (*Qsns.uwa.1AS* and *Qsns.uwa.4AL.1)* from Synthetic W7984 were validated in different genetic backgrounds. The physical position of the surrounding markers seemed to overlap for co-located QTL by CCI reduction and SNS (Table 2). For the QTL on chromosome 1A, the closest flanking marker is *Xgwm33*, 4 cM away from peak QTL location. *Xgwm33* has greater additive effect and therefore was used to validate the QTL effect. For QTL on the long arm of chromosome 4A, the peak marker, *Xbarc343*, with greater additive effect was used to validate the QTL effect. The parental lines of two populations, Synthetic W7984 × Westonia and Synthetic W7984 × Lang were polymorphic for both *Xgwm33* and *Xbarc343.* Synthetic W7984 had a null band for *Xgwm33* and the absence of marker (AA) in progenies reduced senescence as evident in Table 4.

**Table 4:** Fragment size of the two SSR markers, with polymorphism among the parental lines (Synthetic W7984, Westonia and Lang) of validation population, related to QTL for metribuzin tolerance

SSR markers	Parental lines	Fragment size (bp)
*Xgwm33*	Synthetic W7984	Null
	Westonia	142
	Lang	138
*Xbarc343*	Synthetic W7984	201
	Westonia	159
	Lang	159

The validation lines possessing different alleles of markers *Xgwm33* and *Xbarc343* were separated into allele group 1 and allele group 2 using LabChip®. The fragment sizes in Table 4 were used to score the randomly selected lines (24 lines) of validation population into different groups based on the allele groups. For both QTL, genotypes with homozygous alleles from Westonia and Lang had significantly higher (*P* < 0.05) senescence than genotypes with homozygous alleles from Synthetic W7984 (Table 5). For *Xgwm33,* the average senescence of Synthetic W7984 × Westonia and Synthetic W7984 × Lang progenies declined by 28.5 and 28.8%, respectively. Similarly, for *Xbarc343*, the average senescence of Synthetic W7984 × Westonia and Synthetic W7984 × Lang progenies declined by 24.5 and 9.6% respectively (Table 5).

**Table 5:** Validation of the two quantitative trait loci (QTL) in structured and unstructured recombinant inbred line (RIL) populations with the corresponding senescence effect

Co-located QTL for CCI and SNS	RIL population	Marker	AA	aa	*P* value^b^	Effect (%)^c^
*Qcci.uwa.1AS* and *Qsns.uwa.1AS*	Synthetic W7984 × Westonia	*Xgwm33*	4.0^a^	5.6	0.03*	28.5
	Synthetic W7984 × Lang	*Xgwm33*	4.2^a^	5.9	0.01**	28.8
*Qcci.uwa.4AL.1* and *Qsns.uwa.4AL.1*	Synthetic W7984 × Westonia	*Xbarc343*	4	5.3	0.04*	24.5
	Synthetic W7984 × Lang	*Xbarc343*	4.7	5.6	0.02*	9.6

*AA* homozygous alleles from Synthetic W7984, *aa* homozygous alleles from Westonia and Lang

^a^Phenotypic effect for null bands

^b^Student’s t-test (*P* < 0.05) was used to identify differences between the lines of population with distinct allele peaks; **, significant at *P* < 0.01; *, significant at *P* < 0.05

^c^Average decrease in senescence

### Potential candidate genes

One LOD drop-off interval was used to pick the functional markers for a QTL on the current genetic map of Synthetic W7984 × Opata 85 hosted in GrainGenes. For the co-located QTL, *Qcci.uwa.1AS* and *Qsns.uwa.1AS*, six markers are mapped including the tightly linked markers, *Xgwm136* (left) and *Xgwm33* (right) with one-LOD drop off interval between 3.9 and 17.3 cM and 3.9 and 14.3 cM, respectively (Fig. 2). The BLAST search of markers on the wheat genome identified genes with known functions on the URGI-Jbrowse database. This includes uncharacterized gene involving oxidoreductase activity, interestingly related to metabolic detoxification/Xenobiotic degradation (Table 6).

**Table 6:** List of potential candidate genes or proteins related to photosynthesis and metabolic detoxification related to the three major quantitative trait loci (QTL)

QTL name	UniProtKB Gene ID	Length (bp) and direction	Subcellular location	Molecular function	Biological process
*Qcci.uwa.1AS* and *Qsns.uwa.1AS*	A0A1D6RPK4_WHEAT	3063+	–	Oxidoreductase activity	Catalysis of oxidation-reduction reaction
*Qcci.uwa.2DS* and *Qsns.uwa.2DS*	W5C9Y4_WHEAT	840–	Chloroplast	Ribulose-bisphosphate carboxylase activity Monooxygenase activity	Photosynthesis Carbon fixation Photorespiration
	A0A1D5W3W9_WHEAT	989+	–	Glycosyltransferase activity	Metabolic detoxification/Xenobiotics degradation
	A0A1D5X620_WHEAT	1689–	Cytoplasm	Phosphotransferase activity ATP binding Kinase activity	Metabolic and transcriptional processes
*Qcci.uwa.4AL.1* and *Qsns.uwa.4AL.1*	A0A1D5VWT5_WHEAT	2302–	–	Serine/threonine-specific protein kinase (transferase activity) ATP binding	Metabolic and Post-translation modification

+/− Sign indicates the direction (forward/reverse) on the strand; bp indicates base pairs

For QTL, *Qcci.uwa.2DS* and *Qsns.uwa.2DS*, six flanking markers were mapped in close vicinity, including the tightly mapped markers, *Xgwm210* (left) and *Xgwm484* (right) with one-LOD drop off interval between 18.3 and 27.8 cM and 18.3 and 29.7 cM for CCI reduction and SNS, respectively (Fig. 2). A blastN search against *Triticum aestivum* TGACv1 (genomic sequence), identified three potential genes— *rbcs* (ribulose biphosphate carboxylase small chain), *glycosyltransferase* and *phosphotransferase* — involved in the metabolic exclusion of Xenobiotics (Table 6).

QTL, *Qcci.uwa.4AL.1* and *Qsns.uwa.4AL.1* had 13 markers on the genetic map of Synthetic W7984 × Opata 85 within the support intervals of 48.6 to 56.7 cM and 49.6 to 58.3 cM by CCI reduction and SNS, respectively (Fig. 2). The blastN search of markers onto *Triticum aestivum* TGACv1 (genomic sequence) identified a potential candidate gene that has a known function in Xenobiotic degradation. These include a gene which codes for Serine/threonine-specific protein kinase and ATP binding protein (Table 6).

## Discussion

Chlorophyll content index and senescence were highly correlated, reflected in the mapped QTL, and both phytotoxic traits had identical linkage groups and location. The moderate to high levels of broad-sense heritability indicate that the high proportion of genetic variability was measured by chlorophyll traits in the mapping population. A cascade of reactions occur in cells that encounter herbicides and this, in turn, reduces the net photosynthetic rate due to the production of active oxygen species. Any reduction in fundamental processes such as photosynthesis may have a negative effect, which would extend beyond photosystem II (PSII) to cause a down-regulation of total carbon gain causing imbalance between the rate of photo-damage to PSII and the rate of the repair of damaged PSII, reducing plant yield. A higher value of CCI reduction and SNS indicates that a proportion of PSII reaction centers have been damaged. This phenomenon is observed in plants under stress conditions [14].

By single-locus CIM analysis, four QTL for metribuzin tolerance were mapped (Table 2). Map positions of multiple QTL detected on the long arm of chromosome 4A, within 10 cM map distance, validated by fitting multiple regression model using the MIM method of QTL Cartographer. It helped to eliminate false positive QTL and get realistic estimates of the total variation explained by the QTL because the R^2^ value of the multiple QTL model takes into account their lack of independence [15, 16]. The refinement of map-positions was possible due to good marker density of the regions, which enhanced the mapping resolution around these tolerance loci.

Three significant genomic regions on chromosome 1A, 4A and 2D associated with metribuzin tolerance, explained phenotypic variation of 10–19% and 8–20% using CCI reduction and SNS respectively, signifying that herbicide tolerance is a genetically governed quantitative trait that reflects the cultivar’s ability to withstand herbicide at the level of PSII. Similarly, Javid et al. [8] reported QTL for metribuzin tolerance in field pea (*Pisum sativum* L.) with phenotypic variance ranging from of 12–21%. Several pleiotropic QTL affecting leaf senescence and grain yield and/or grain protein concentration were identified on group 2 and group 7 chromosomes in wheat [17]. The QTL(s) for metribuzin tolerance with LOD > 3.0 was detected from both Synthetic W7984 and Opata 85 parent which suggests that adaptive traits in both Synthetic W7984 and Opata 85 could harbour a suite of herbicide tolerance-related adaptive features or tolerance could be due to new gene/allele combination that should be further explored. However, the QTL (*Qcci.uwa.2DS*) detected from Opata 85 could not be validated due to the lack of validation population.

In the present study, all the QTLs detected by CCI reduction co-locate with senescence which supports the hypothesis that a non-redundant function of relative leaf chlorophyll content and cellular oxidative processes causes senescence. Co-mapping of QTL for correlated traits may result from tight linkage of several genes [18] or the pleiotropic effect of major genes [19]. Zhang et al. [12] elucidated the linear relationship between chlorophyll content and yield by co-located QTL for both traits reflecting pleiotropism. Fontaine et al. [20] reported co-localization of senescence with agronomic traits such as grain number per spike, the amount of protein per grain, and thousand kernel weight in common wheat. Kichey et al. [21] confirmed a positive correlation between chlorophyll content (visual senescence) and grain yield, and total nitrogen content. Similarly, Gallais and Hirel [22] reported non-redundant function between the activities of two enzymes—glutamine synthetase and glutamate dehydrogenase—and yield-related traits. In wheat, consistent co-localized QTL have been reported and QTL for correlated traits have been mapped together [20, 23].

The two major QTL located on short and long arm of wheat chromosome 1A and 4A— *Qsns.uwa.1AS and Qsns.uwa.4AL.1*—from Synthetic W7984 positively contributing to metribuzin tolerance was validated in different genetic background. Validation of functional marker for its phenotypic effects in different genetic backgrounds/environments under herbicide– treated conditions is essential to rule out statistical errors/artefacts [24] before applying in marker-assisted selections. There are numerous markers present within QTL support interval and use of conventional bench-top molecular techniques to check polymorphism and band size is time-consuming, lacks specificity, and is labor-intensive. High-throughput genotyping of markers using LabChip® for marker validation has enabled improved read-out of information from molecular systems using computer chips.

Numerous nuclear and cytoplasmic genes were identified by blasting the markers, within the support interval, against the wheat genome. This corroborates with the findings of Ratliff et al. [4] which stated that metribuzin tolerance is a complex trait controlled by both nuclear and cytoplasmic genes. Nevertheless, amidst numerous genes having unknown functions, putative functions, and hypothetical/uncharacterized proteins, it is promising that the identified QTL also hold some genes directly or indirectly associated with photosynthesis, in particular, the *rbcS* gene which codes for Rubisco protein involved in photosynthetic CO_2_ fixation. Rubisco is the most abundant protein, accounting for 12–35% of total leaf N in plants [25, 26]. QTL on short arm of 2D chromosome has been found closely associated to one of the photosynthesis-related genes, *rbcS*. Group 2 and Group 5 chromosomes in wheat has been reported to harbour multigene family of *rbcS* [27]. Genes involved in photosynthesis and regulation of senescence associated protein has been found responsive to metribuzin tolerance in wheat [28].

The identified QTL regions are an ideal target for the characterization of a gene(s) underlying this locus. The identified chloroplast gene (*rbcS*) could have a direct impact on maintaining high net photosynthetic rate during herbicide stress. The identified glycosyltransferases (GT) gene is involved in detoxification of a variety of toxic chemicals, including pollutants and herbicides in Phase II herbicide detoxification [29–32]. The conjugation reactions enable GT to diversify the secondary metabolites via sugar attachments to maintain cell homeostasis by quickly and precisely controlling plant hormone concentration, and detoxify herbicides by adding sugars onto molecules [33]. In conclusion, the proteins encoded by the identified genes are involved in the oxygen-evolving complex, and repair of the PSII complex and xenobiotic detoxification. However, since numerous biological processes are associated with these candidate genes, more detailed experimental analyses will be needed to confirm their roles in metribuzin tolerance.

Integrated effective weed control is vital for economical food production. In dry land farming, particularly those with Mediterranean climate, wheat and broad-spectrum weeds actively grow throughout the wet winter wheat growing season. Australia has the second highest reported occurrence of herbicide-resistant weeds worldwide, which is the key agronomic issue for the farmers. Making direct selections for chlorophyll traits will positively influence herbicide tolerance and grain yield. SSR markers, *Xgwm136 and Xbarc343* validated in different genetic background can be used for marker-assisted selection to potentially reduce phytotoxic effects of herbicide and improve photosynthetic efficiency and yield. Consequently, the identified and validated favourable alleles could be introgressed into elite wheat cultivars used in dryland farming for sustainable wheat production. This will be a more effective strategy to control weeds without compromising wheat productivity in dry-land farming of Australia and worldwide.

## Conclusion

To our knowledge, this is the first report on identification of QTL and functional markers associated to herbicide tolerance at seedling stage in wheat. This study has shown that CCI reduction and SNS are effective parameters for evaluating metribuzin tolerance in wheat breeding. Metribuzin tolerance is a complex trait and direct phenotypic selection is time-consuming, labour intensive and could be hindered significantly by environmental factors. Marker-assisted selection is a tool for precision plant breeding and offers several advantages over direct phenotypic screening. Identification of genomic regions responsible for metribuzin tolerance followed by validation of closely linked markers in two populations, specifically segregating for chlorophyll traits, identified genes directly or indirectly affecting the functionality of PSII and herbicide metabolism in the plant system. The identified flanking markers can be used in marker-assisted selection for breeding metribuzin tolerant wheat. Further, the identified QTL could be fine mapped using high number of transgressive lines to identify the exact candidate genes for the trait or the identified candidate genes could be further investigated/targeted to improve the PSII efficiency during metribuzin stress.

## Methods

### Genetic stocks

One hundred and eleven recombinant inbred lines (RILs) (F_8_ generation) from the international Triticeae mapping initiative (ITMI) mapping population derived from a cross between Synthetic W7984 (*Triticum turgidum* cv. Altar84/*Aegilops tauschii* Coss. line WPI 219) and Mexican spring wheat cultivar Opata 85 [34] were used to identify QTL/genomic regions responsible for metribuzin tolerance. Following the identification and mapping of QTL, crosses between Synthetic W7984 as the common parent and two other genotypes (Westonia and Lang) were used to validate the phenotypic effect of these QTL in other genetic backgrounds. These two F_3.4_ RIL populations (Synthetic W7984 × Westonia and Synthetic W7984 × Lang) were evaluated for one year to validate the QTL identified in the mapping population. SSR markers in close vicinity to the QTL were used to genotype 24 RILs from the crosses mentioned above.

### Experimental design and herbicide treatment

Package DiGGeR [35] was used to generate a row–column design with two blocks. A seedling tray (Rite-Gro) with 6 × 5 cells filled with homogenous river sand was equilibrated with water to 100% capacity. One seed/per cell was sown. Treatment and control trays were sprayed with a differential metribuzin dose of 400 g ai ha^− 1^, dose which differentiates tolerant and susceptible lines as identified in the previous study [10], and water, respectively, perpendicular to the tray surface in two passes at a flow rate of 118 L ha^− 1^ and 200 kPa in a cabinet spray chamber. The trays were maintained in a phytotron, where the temperature was set to 25/15 °C day/night and watered regularly every 48 h. 12 treatment and 12 control trays were randomized across two blocks. The mapping population was assessed in two independent trials, in 2016 and 2017, with three replicates. The same experimental setup and rate of metribuzin were used to evaluate the phytotoxic effects of metribuzin in the validation populations.

### Phenotypic evaluation

The phytotoxic effects of metribuzin in wheat seedlings were recorded using a portable Minolta SPAD-502 chlorophyll meter (Spectrum Technologies, Inc., Plainfield, IL, U.S.). The SPAD chlorophyll content index (CCI) scores were obtained from leaf lamina at 16 day after treatment (DAT) and the average reading from two fully emerged leaves represented the final score. This index is linearly related to chlorophyll concentration [36]. The effect of metribuzin was computed as a percentage of CCI reduction relative to the control. Lower CCI reduction represents higher tolerance and higher CCI reduction represents greater susceptibility. Leaf senescence was visually estimated using a scale of 0 (no senescence/phytotoxicity) to 10 (100% senescence/dead), 16 DAT. This count was defined as the senescence score (SNS), with a low SNS representing less loss of chlorophyll and greater tolerance and a high SNS representing more loss of chlorophyll and greater susceptibility. Control plants were green and healthy with no visual senescence; therefore, senescence was not scored for control plants.

### Molecular markers and linkage map

Molecular marker data and linkage map of the Synthetic W7984 × Opata 85 RIL mapping population were accessed from the GrainGenes database (https://wheat.pw.usda.gov/cgi-bin/GG3/report.cgi?class=mapdata&name=Wheat%2C%20Synthetic%20x%20Opata%2C%20BARC). The chromosomal location for metribuzin tolerance was mapped by integrating appropriate phenotypic and genotypic/segregating data containing a total of 1476 SSR and RFLP markers. The genetic map spanned a length of 3715 cM with a mean marker density of 1 cM, distributed across 21 chromosomes of wheat. Markers with >70% missing data were removed from the dataset.

### Quantitative trait loci analysis

QTL analysis was performed for the trait means using the composite interval mapping (CIM) method of WinQTLcart2.5. Based on 1000 permutations [37], logarithm of odds (LOD) peaks ≥3 were used to declare significant QTL for SPAD CCI reduction and SNS, respectively, on data combined across two years. The contribution rate (R^2^) was calculated as the percentage of variance explained by each QTL in proportion to the total phenotypic variance. QTL were classified as major when the phenotypic variance was more than 10% and minor for less than 10% [38]. A one-LOD drop from the peak position was used as a support interval for each QTL location. Flanking or tightly linked markers were selected within the QTL supportrange. QTL with a negative additive effect for a trait implies alleles from the tolerant parent increase tolerance by decreasing phytotoxicity and positive additive effect mean alleles from the susceptible parent increase tolerance by decreasing phytotoxicity.

Finally, a QTL was declared when the region was mapped with both the CCI reduction and SNS scores. This was to protect against type I errors (finding false positive QTL) and type II errors (missing real QTL). In cases of multiple QTL detected on the same linkage group, inter-locus interaction or epistasis was determined by simultaneous analyses of the QTL in a multiple regression model using the multiple interval mapping (MIM) method of QTL Cartographer.

### Genotyping populations for marker validation

Genomic DNA was extracted from the leaves of three-week-old seedlings of individual plants from the parental lines of Synthetic W7984, Westonia and Lang, and each of the F_3:4_ validation lines from Synthetic W7984 × Westonia and Synthetic W7984 × Lang using the cetyl trimethyl ammonium bromide (CTAB) method and then suspended in T.E. buffer (pH 8.0) for storage and analysis. DNA concentration was assessed by Qubit 2.0 fluorometer using the Qubit ds DNA Broad Range Assay. The PCR reaction mixture (15 μL) containing 100 ng template DNA, 20 μM forward and reverse primers each, and 7.5 μL Taq HS mix (Bioline, NSW, Australia) was amplified in a thermocycler (BIO-RAD). PCR thermal cycling was programmed at: 95 °C for 5 min, 35 cycles of denaturation at 95 °C for 30 s, annealing at temperature obtained from GrainGenes for individual SSR markers for 30 s, elongation at 72 °C for 45 s, and final extension at 72 °C for 5 min. The primers were obtained from Sigma-Aldrich (Sigma-Aldrich Pty Ltd., NSW, Australia). Typically, SSR reactions were multiplexed in pairs based on their annealing temperature and amplicon size. To minimize background signals, PCR products were diluted with nuclease-free water in a 1:15 ratio. The diluted amplicons were analyzed by automated capillary electrophoresis LabChip® GX instrument from Perkin Elmer. A DNA 5 K Perkin-Elmer LabChip® which gives resolution from 100 to 5000 bp, was used to separate the bands with appropriate sizing. The microfluidic 5 K chip was loaded with gel and marker in the specified containers according to the manufacturer’s protocol. PCR plates were centrifuged (5 min, 2000 rpm) and placed without caps or adhesive film in the electrophoresis system. The chip was primed for 5 min, and PCR samples were injected into the chip’s micro-capillaries connected to electrodes. Each sample, mixed automatically with an internal marker, was run through these micro-capillaries according to their size and nucleotide composition. The DNA fluorescence measurement was recorded as a function of time on an electropherogram and the DNA ladder defined the product sizes. Primary data (electropherograms) were then converted to virtual gels by the machine software for the analyses. LabChip Reviewer 5 Software was used to analyze peaks, percent purity and molecular weights. Settings were kept constant for all runs to make comparisons within and between runs.

Phenotypic effects of major QTL alleles detected from Synthetic W7984 were estimated in different genetic background using closely linked markers. Recombinant inbred lines of two F_3.4_ RIL populations (Synthetic W7984 × Westonia and Synthetic W7984 × Lang) carrying the same QTL alleles were grouped. The mean phenotypic performance of genotypes based on the two types of allele combinations (AA and aa) were compared using Fisher’s protected least significant difference (LSD) at *P* = 0.05. The phytotoxic effect was computed as the mean SNS of homozygous dominant (AA) relative to homozygous recessive (aa) allele.

### Statistical analysis

The phenotypic data analyses were performed using GenStat statistical software 17th edition (VSN International 2014). Analysis of variance (ANOVA) was conducted for unbalanced design based on the following model: P_*ij*_ = μ + g_*i*_ + y_*j*_ + gy_*ij*_ + Ɛ_*ijk*_, where P_*ij*_ is the observed phenotypic mean; μ is the overall/population mean; g_*i*_ is the effect due to the i^th^ genotype; y_*j*_ is the effect due to j^th^ year; Ɛ_*ijk*_ is the random error. The adjusted mean was further used to calculate the percentage CCI reduction. Variance components and broad-sense heritability (*h*^*2*^) were estimated from the ANOVA, as in He et al. [39]. Heritability was estimated using the formula: h^2^ = ${\sigma}_g^2/$(${\sigma}_g^2+$ ${\sigma}_{gy}^2/y$ + ${\sigma}_e^2/ ry$) for multiple years where ${\sigma}_g^2$ is the genetic variance; ${\sigma}_{gy}^2$ is the variance for genotype by environment interaction; ${\sigma}_e^2$ is the residual variance; y is the number of years and r is the number of replications. The estimated genotypic interaction and error variances were calculated as: ${\sigma}_g^2=\frac{MSg- MSgy}{\mathrm{ry}}$; ${\sigma}_{gy}^2=\frac{MSgy- MSe}{\mathrm{r}}$ and ${\sigma}_e^2= MSe$

### Identification of potential candidate genes

The physical position of the flanking markers was identified in the GrainGenes wheat database (https://wheat.pw.usda.gov/cgi-bin/GG3/browse.cgi?class=marker). The QTL support range was estimated based on one LOD drop off interval on identified genomic regions. The marker sequences within the QTL support range were blasted against the Ensembl Plants wheat (http://plants.ensembl.org/Triticum_aestivum/Info/Index) to find the Traes numbers of the genes. The Traes numbers were searched in the UniProt in TrEMBL (http://www.uniprot.org/?-id+2fYRW1ChXSa+-fun+Pagelibinfo+-info+TREMBL) to obtain more information including protein domain, family, molecular and biological functions of the potential candidate genes. Only those genes with known function and/or related to photosynthesis and metabolic detoxification were counted as potential candidate genes. The graphical representations of QTL on linkage groups were drawn by MapChart 2.2 software [40].

## Additional files

Additional file 1: **Table S1.** Mean percentage chlorophyll reduction measured across two years (2016 and 2017). (XLSX 22 kb)

Additional file 2: **Table S2.** Mean senescence (visual injury) measured across two years (2016 and 2017). (XLSX 17 kb)

## Abbreviations

CCI: Chlorophyll content index
CIM: Composite interval mapping
MAS: Marker-assisted selection
MIM: Multiple interval mapping
PS II: Photosystem II
QTL: Quantitative trait locus
RIL: Recombinant inbred lines
SNS: Senescence
